# The Da Vinci Single-Port (SP) Treatment of Nutcracker Syndrome: A Case Report of a Novel Approach

**DOI:** 10.7759/cureus.58447

**Published:** 2024-04-17

**Authors:** Mohammed Abuzenada, Seokhwan Bang, Sung-Hoo Hong

**Affiliations:** 1 Department of Urology, Seoul St. Mary's Hospital, The Catholic University of Korea, Seoul, KOR; 2 Department of Urology, The Catholic University of Korea, Seoul, KOR

**Keywords:** extravascular stent, single port robotic-assisted surgeries, da vinci sp, minimally invasive surgeries, nutcracker syndrome

## Abstract

Nutcracker syndrome (NCS) is a rare disease affecting the left kidney. Surgical management is the only choice of treatment. Minimal invasive surgeries can be effective and may prevent complications of the major surgery.

We present the case of a 33-year-old woman suffering from chronic left flank pain, diagnosed with NCS and treated with extravascular stents. Robotic-assisted extravascular stent insertion was performed using the Da Vinci single-port (SP) (Intuitive Surgical, Inc., Sunnyvale, USA) system.

This approach offers the advantages of minimal invasiveness, precise stent placement, and reduced operative time. To our knowledge, this is the first case of using Da Vinci SP for this indication. Further studies are needed to evaluate the long-term outcomes and safety.

## Introduction

Nutcracker syndrome (NCS) is a rare syndrome where the left renal vein (LRV) gets trapped between the abdominal aorta and superior mesenteric artery (SMA) and causes left flank pain, hematuria, left-side varicocele, and ovarian vein syndrome [1]. Surgery is usually the standard treatment for NCS, with an open vessel transposition of the renal vein or an autotransplantation being the most commonly used method [2]. The practice of minimally invasive surgery has increased due to the associated morbidity and the potential for serious complications [3]. This study aims to introduce a novel technique for extravascular stenting using a single-port robotic-assisted procedure.

## Case presentation

A 33-year-old woman presented to the outpatient clinic with recurrent attacks of left flank pain and pelvic pain accompanying persistent microscopic hematuria for several years. There is no evidence of a urinary tract infection or history of kidney stones. The patient underwent further investigations. Hemoglobin level, white cell count (WBC), platelets, and renal profile were within normal range. The urine culture was negative for bacterial growth. Urine analysis showed RBC 4-9, no WBC, or nitrate.

**Figure 1:**
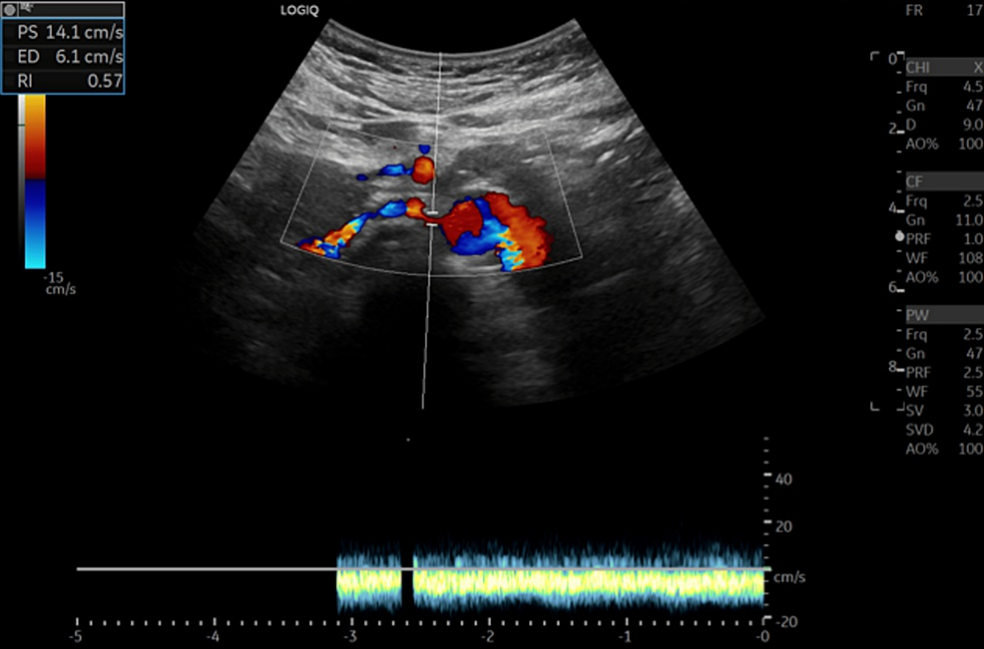
The figure shows a color Doppler ultrasound image of the aortomesenteric portion of the LRV displaying a bright color flow signal. The signal is attributed to aliasing artifacts resulting from the high flow velocity in the area. LRV: left renal vein

**Figure 2:**
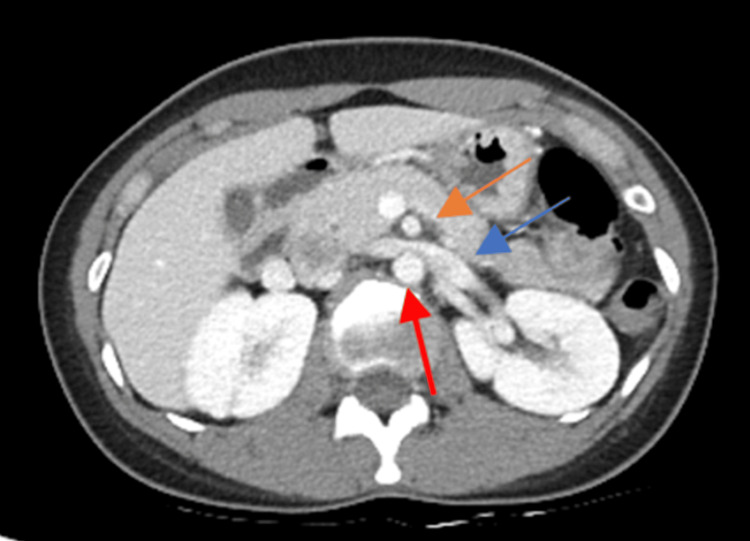
The figure shows the axial view of a computed tomography image of the abdomen and pelvis with intravenous contrast, revealing the compression of the LRV between the AA and SMA. The image is annotated with a red arrow indicating AA, an orange arrow indicating SMA, and a blue arrow indicating LRV. LRV: left renal vein; AA: abdominal aorta; SMA: superior mesenteric artery

Color Doppler US of LRV shows a bright color flow signal at an aortomesenteric portion of LRV due to aliasing artifacts caused by high-flow velocity, possibly suggesting NCS (see Figure 1). CT abdomen and pelvic with IV contrast showed compression of LRV between the aorta and SMA (see Figure 2).

**Figure 3:**
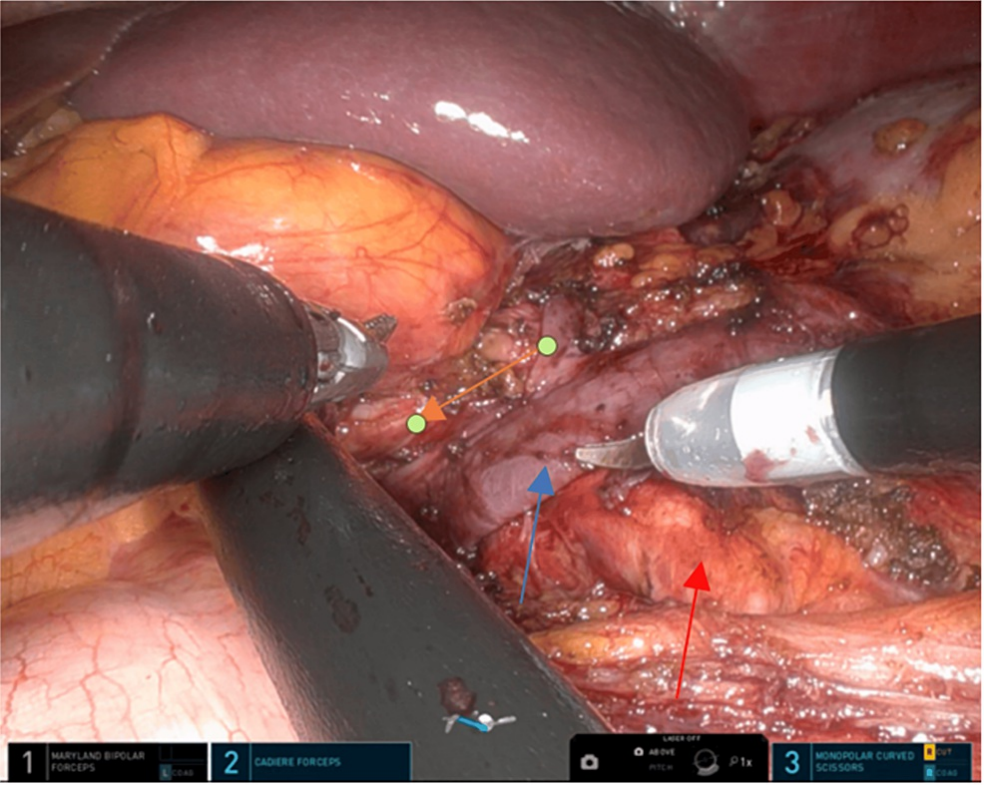
The figure displays the dissection of the LRV until its junction with the IVC, indicated by a blue arrow in the image. The red arrow represents AA, while the orange arrow depicts the SMA. LRV: left renal vein; AA: abdominal aorta; SMA: superior mesenteric artery; IVC: inferior vena cava

**Figure 4:**
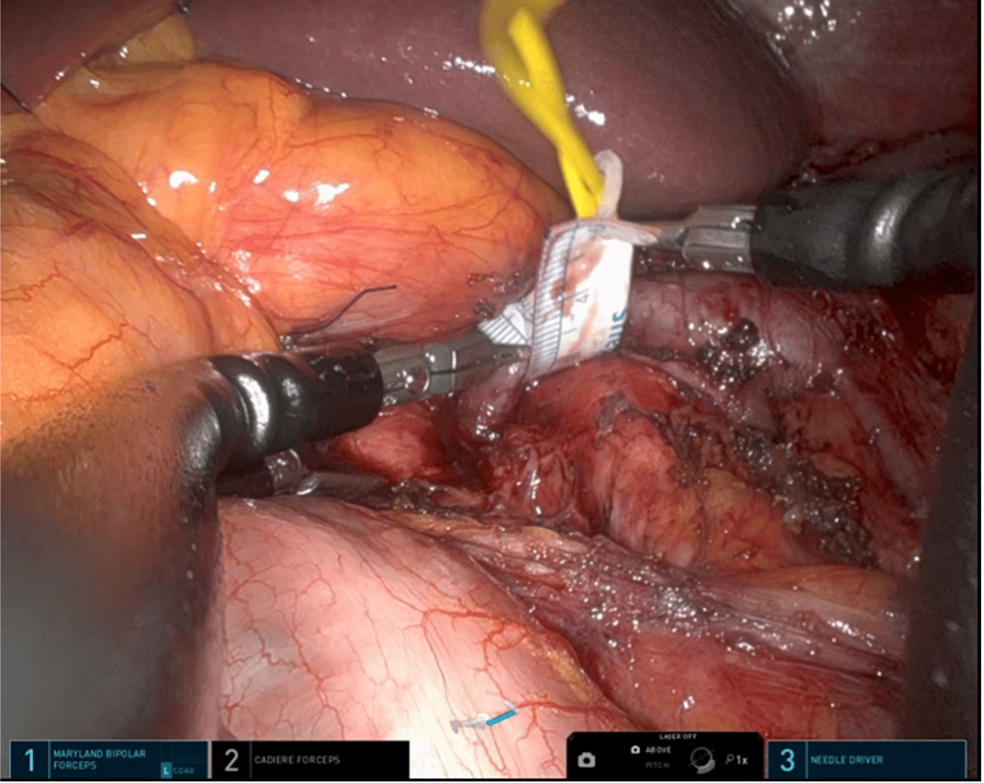
The figure displays the intraoperative measurement of the diameter of the left renal vein (LRV) during surgery. The diameter of the LRV was measured using a caliper and recorded in millimeters. This figure provides a visual representation of the measurement process and highlights the importance of accurate measurement of the LRV diameter during surgery.

**Figure 5:**
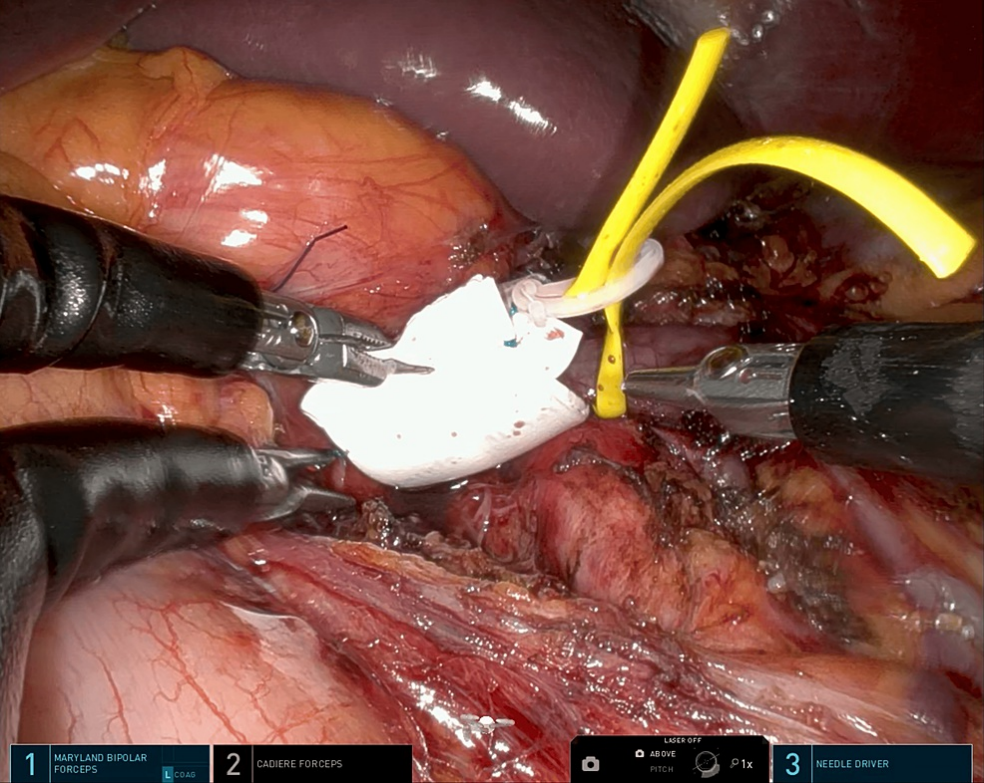
The figure illustrates the reinforcement of a PTFE stent over the LRV. PTFE: polytetrafluoroethylene; LRV: left renal vein

**Figure 6:**
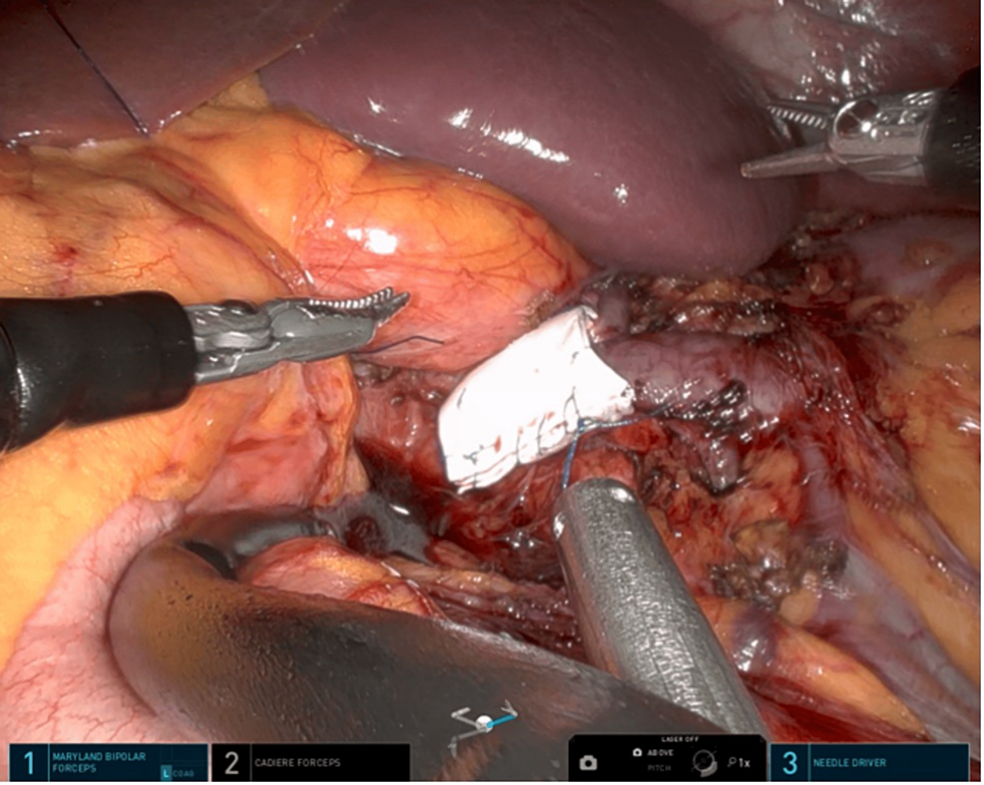
The figure shows that an interrupted suture was performed to prevent migration of the stent.

The patient underwent the Da Vinci single-port (SP) (Intuitive Surgical, Inc., Sunnyvale, USA) robotic-assisted extravascular stent insertion in the LRV under general anesthesia. A 4 cm incision was made near the umbilicus, and a floating docking technique was used. The retroperitoneal space was accessed through a transperitoneal approach, and the LRV was exposed up to its junction with the inferior vena cava, SMA, and abdominal aorta (see Figure 3). The LRV diameter was measured intraoperatively (see Figure 4), and an externally reinforced polytetrafluoroethylene (PTFE) graft (GORE-TEX®; W. L. Gore & Associates, Inc., Newark, USA) was placed over the LRV (see Figure 5). The stent was secured with interrupted sutures to prevent migration (see Figure 6). The console time was 74 minutes, and blood loss was minimal. The patient recovered well and was discharged home after three days. The patient reported no symptoms at the follow-up visit. A CT scan after six months showed no stent migration and resolution of the previous LRV dilation.

## Discussion

NCS occurs when the LRV becomes compressed between the aorta and the SMA, resulting in left flank pain, hematuria, left-sided varicocele, or ovarian vein syndrome [4]. Surgical treatment for NCS typically involves open vessel transposition of the renal vein or SMA. However, this procedure is associated with high complication rates, including bleeding, thrombosis, and paralysis [5]. Thrombosis of the SMAs can be a serious consequence of this surgery [6]. An alternative treatment option is endovascular stenting of the renal vein, which is safe, easy, and minimally invasive, with a good outcome. Still, it requires long-term antiplatelet medication to prevent one of its complications such as blood clots. A laparoscopic extravascular stent is another option that is similar in outcome to endovascular stenting [7]. Robotic-assisted extravascular stenting had a similar outcome compared to the laparoscopic approach, with a reduced risk of lymphatic complications [8].

## Conclusions

Robotic-assisted extravascular stenting of the LRV appears to be safe, effective, and cosmetically appealing, and it avoids major complications caused by the transposition of major vessels. The effectiveness of this technique will, however, require long-term follow-up.
